# Predictive value of long-term changes of growth differentiation factor-15 over a 27-year-period for heart failure and death due to coronary heart disease

**DOI:** 10.1371/journal.pone.0197497

**Published:** 2018-05-17

**Authors:** Nina Fluschnik, Francisco Ojeda, Tanja Zeller, Torben Jørgensen, Kari Kuulasmaa, Peter Moritz Becher, Christoph Sinning, Stefan Blankenberg, Dirk Westermann

**Affiliations:** 1 Department of General and Interventional Cardiology, University Heart Center Hamburg-Eppendorf, Hamburg, Germany; 2 DZHK Affiliation, partner site Hamburg/Kiel/Lübeck, Germany; 3 Research Center for Prevention and Health, Glostrup University Hospital, Glostrup, Denmark; 4 Department of Public Health, Faculty of Health and Medical Science, University of Copenhagen, Copenhagen, Denmark; 5 Faculty of Medicine, Aalborg University, Aalborg, Denmark; 6 National Institute for Health and Welfare, Helsinki, Finland; Medizinische Hochschule Hannover, GERMANY

## Abstract

**Background:**

Growth differentiation factor-15 (GDF-15), Cystatin C and C-reactive protein (CRP) have been discussed as biomarkers for prediction of cardiac diseases. The aim of this study was to investigate the predictive value of single and repeated measurements of GDF-15 compared to Cystatin C and CRP for incidence of heart failure (HF) and death due to coronary heart disease (CHD) in the general population.

**Methods and results:**

Levels of GDF-15, CRP and Cystatin C were determined in three repeated measurements collected 5 years apart in the DAN-MONICA (Danish-Multinational MONitoring of trends and determinants in Cardiovascular disease) cohort (participants at baseline n = 3785). Cox regression models adjusted for cardiovascular risk factors revealed significantly increased hazard ratios (HR) for GDF-15 for incident HF 1.36 (HR per interquartile range (IQR) increase, 95% confidence interval (CI): 1.16; 1.59) and for death from CHD 1.51 (HR per IQR increase, 95% CI: 1.31, 1.75) (both with p<0.001). Joint modeling of time-to-event and longitudinal GDF-15 over a median 27-year follow-up period showed that the marker evolution was positively associated with death of CHD (HR per IQR increase 3.02 95% CI: (2.26, 4.04), p < 0.001) and HF (HR per IQR increase 2.12 95% CI: (1.54, 2.92), p<0.001). However using Cox models with follow-up time starting at the time of the third examination, serial measurement of GDF-15, modeled as changes between the measurements, did not improve prediction over that of the most recent measurement.

**Conclusions:**

GDF-15 is a promising biomarker for prediction of HF and death due to CHD in the general population, which may provide prognostic information to already established clinical biomarkers. Repeated measurements of GDF-15 displayed only a slight improvement in the prediction of these endpoints compared to a single measurement.

## Introduction

The prevalence of heart failure (HF) is steadily increasing with the growing elderly population with a current prevalence of over 23 million worldwide [1]. Simultaneously, cardiovascular diseases (CVD) remain the number one cause of death in Europe although the incidence of coronary heart disease (CHD) has declined [2]. The use of circulating biomarkers has improved risk estimation of HF and CVD in apparently healthy individuals [3, 4]. The N-terminal pro-B-type natriuretic peptide (NT-proBNP), which is released due to myocardial stress and cardiac volume overload, has become one of the most important biomarkers in diagnosis and prognosis of HF [4]. With the advent of novel biomarkers and their combinations, the prognostic value and their effect in the primary prevention in patients suffering from CVD or HF are currently discussed. Growth differentiation factor-15 (GDF-15), also known as macrophage inhibitory cytokine-1 (MIC-1), is a member of the transforming growth factor (TGF-ß) cytokine superfamily, which has been examined as a novel emerging biomarker in HF, cancer and atherosclerotic diseases including CVD [5–10]. Cardiac expression of GDF-15 has been induced in response to oxidative stress, ischemia/reperfusion injury, inflammation, stress induced by biomechanically stretching in the heart and pressure overload [11–13]. Nonetheless, in community-based populations, Brown and colleagues first described in a prospective case-control study that increased plasma levels of GDF-15 in healthy women were accompanied with a higher risk for future cardiovascular events [14]. In addition, several subsequent studies revealed that increased levels of GDF-15 are associated with increased cardiovascular mortality and all-cause mortality independent of classical cardiovascular risk factors in community-dwelling individuals [8, 15–17]. In 2012 the Framingham Heart Study first showed that GDF-15 is strongly related with the risk of HF and death in a community based setting with 3,428 participants [18].

The aim of the present study was to investigate the prognostic value of a single measurement of GDF-15 for prediction of the development of HF and death due to coronary heart disease (CHD) in the general population. Moreover, we examined whether long-term changes (repeated measurements) of GDF-15, could provide additional prognostic information in this study population. For that purpose, comprehensive data of the DAN-MONICA Cohort (Danish-Multinational MONitoring of trends and determinants in Cardiovascular disease) was analyzed with a median follow-up of 27 years of more than 3500 individuals.

## Methods

### Study population

The present study included the DAN-MONICA I cohort, using data from the BiomaCaRE (Biomarker for Cardiovascular Risk Assessment in Europe) project [19]. This prospective population-based cohort is derived as a random sample of the population in eleven municipalities in Copenhagen County, Denmark [20]. Random sampling is based on the national population register, stratified by gender and year of birth. The cohort was linked to the National Hospital Discharge Register, to the Causes of Death Register and the Civil Registration System using the unique personal identification number in Denmark. All participants gave informed consent. The cohort consists of men and women aged 30–60 years having two repeated measurements of baseline risk factors. Round 1 of this cohort constitutes the baseline examinations carried out in 1982–84 (n = 3785). Participants were re-examined in 1987–1988 (Round 2, n = 2987) and 1993–1994 (Round 3, n = 2656). The cohort was followed up until December 2009 using linkage to the National Cause of Death Register and National Hospital Discharge Register. A hospitalization or death with ICD-8 code 427.0, 427.1 or 428, or ICD-10 code I11.0, I13.0, I13.2 or I50 was considered to indicate heart failure. The cause of death due to CHD was coded by ICD-8 code 410–414 or ICD-10 code I20-25. The study was approved by the local ethics committee in Copenhagen County (Københavns Amt, Den videnskabsetiske komite; No: 1980-272-2 1051 NE7bt 2-16-2/43 (85) KA 90238).

The endpoints were defined as first diagnosis of HF and death due to CHD.

### Laboratory methods

Plasma and serum samples were separated from venous blood and stored at -20°C in Glostrup from 1986/1987 and since 2010 in the MORGAM/BiomarCaRE laboratory (Hamburg) at -80°C. All biomarkers were measured in the MORGAM/BiomarCaRE laboratory. GDF-15 (pg/mL) was measured in serum using the Abbott ARCHITECT GDF-15 assay with measurement range between 0–5000 pg/ml and a lower limit of detection (LOD) of 5 pg/. The inter-assay coefficient of variation (CV) was 4.68%, the intra-assay CV was 2.20% mL assessed at a GDF-15 level of 500 pg/mL. Creatinine (mg/dl) was measured using the Abbott ARCHITECT Creatinine enzymatic assay. Estimated glomerular filtration rate (eGFR) was computed using the creatinine based CKD-EPI formula. CRP was measured using the Abbott ARCHITECT CRP vario assay with the measurement ranges of 0.01–16 mg/dl and a LOD of 0.01 mg/dL. The CV was determined at 0.5 mg/dl. The inter-assay had a CV of 4.71%, the intra-assay CV of 0.50%. Cystatin C was measured using the Abbott Cystatin C ARCHITECT c8000 assay with measurement ranges 0.05–8.33 mg/L and a LOD of 0.05 mg/l. The inter-assay and intra-assay CV was 3.97% and 1.27% assessed at a Cystatin C level of 0.71 mg/L, respectively.

### Statistical analysis

Baseline characteristics are given as absolute and relative frequencies for categorical variables, mean and standard deviation or quartiles for continuous variables.

Multiple imputation was used to deal with missing data. Twenty imputed datasets were produced using chained equations [21]. Predictive mean matching was used for all variables and time-to-event information was incorporated into the imputation model. Separate imputations were produced for men and women.

First, the association of the first measurements (round 1) of the biomarkers of interest (GDF-15, CRP and Cystatin C) with death due to CHD and incident HF was examined. The biomarkers were categorized using their quartiles and survival curves were produced by the Kaplan-Meier method. The logrank test was performed to compare the survival curves defined by the biomarker fourths. Afterwards, Cox regressions adjusted for sex, overweight (BMI > 25 kg/m^2^), systolic blood pressure, diabetes, daily smoker, renal insufficiency (eGFR < 60 mL/min for 1.73m^2^) were computed. Age was used as the time scale in the Cox models. To quantify the discrimination of the 25-year event probabilities derived from these Cox models the C-index was computed. To obtain these probabilities the Breslow estimator of the baseline survival function was used. 10-fold cross-validation was used to avoid over-optimism resulting from assessment of the model performance on the same data in which the model was derived [22].

To examine the association of outcome to the longitudinal measurements of the biomarkers, a joint model for the longitudinal marker and time to event was computed [23, 24]. The joint model consists of two parts, a linear mixed effect model for the biomarker and a proportional hazards model for the survival part. For each marker, the linear mixed effect model included a random intercept and a random slope from time since baseline for each individual. Sex, age at round 1 (cross-sectional effect of age), time since baseline (longitudinal effect of age), overweight, systolic blood pressure, LDL cholesterol, diabetes, daily smoker and renal insufficiency were used as fixed effects in the mixed effect model. The survival part of the joint model used age as the time scale and was adjusted for the same covariates as was the Cox model described in the previous paragraph. For the latter model the values of the covariates were updated at each round.

To assess how changes in biomarker values may improve predictive performance over that of the most recent measurement, the available marker history up to the third examination (round 3) was modelled in different ways. The difference between the values at round 3 and round 2 (round 1 respectively) was considered and the round 3 measurement was also used. These variables entered a Cox model with follow-up time starting at round 3. All models used the same adjusted covariates as the Cox regressions described earlier and age was used as the time scale. The models including the marker differences as predictors where further adjusted for the round 3 measurement of the corresponding marker. The C-index was calculated to examine the discrimination of the marker trend over the last available marker measurement (at round 3).

For all regression analyses the biomarkers were used after being log-transformed and their hazard ratios are presented per interquartile range (IQR) increase, where the IQR is defined as the distance between the 25^th^ and 75^th^ percentile of the log-transformed marker. Analyses were performed with R version 3.3.0 [25]. A more detailed description is provided in the S1 File.

## Results

### Baseline characteristics

The baseline characteristics of the imputed datasets of the patients for each round are shown in Table 1. Baseline characteristics of the nonimputed dataset are shown in S1 Table.

**Table 1:** Baseline characteristics of the DAN-MONICA cohort according to round 1–3 (Data for the imputed datasets).

	round 1 (n = 3785)	round 2 (n = 3672*)	round 3 (n = 3461*)
**Examination age (years)**	45.5±11.0	50.2±11.0	55.6±10.9
**Male (%)**	1940.0 (51.3)	1867.0 (50.8)	1726.0 (49.9)
**BMI (kg/m**^**2**^**)**	24.6±3.9	25.2±4.0	26.0±4.3
**BMI>25 kg/m**^**2**^ **(%)**	1524.0 (40.3)	1696.4 (46.2)	1867.8 (54.0)
**HDL cholesterol (mmol/L)**	1.5±0.4	1.5±0.4	1.4±0.4
**LDL cholesterol (mmol/L)**	3.7±1.1	4.1±1.1	4.1±1.1
**Total cholesterol (mmol/L)**	5.8±1.2	6.1±1.2	6.2±1.2
**Systolic BP (mmHg)**	123.3±16.8	126.6±19.1	130.0±19.6
**Diastolic BP (mmHg)**	77.2±10.9	81.8±10.6	82.3±10.8
**Daily smoker (%)**	1768.0 (46.7)	1824.8 (49.7)	1362.5 (39.4)
**Diabetes No. (%)**	86.0 (2.3)	122.9 (3.3)	153.9 (4.4)
**eGFR (mL/min for 1.73m**^**2**^**)**	109.4 (96.8, 119.9)	104.1 (92.5, 113.5)	97.3 (85.3, 107.1)
**eGFR<60 mL/min for 1.73m**^**2**^ **No. (%)**	78.5 (2.1)	70.0 (1.9)	111.7 (3.2)
**Creatinine (mg/dL)**	0.8 (0.7, 0.9)	0.8 (0.7, 0.9)	0.8 (0.7, 1.0)
**Cystatin C (mg/L)**	0.7 (0.6, 0.8)	0.7 (0.7, 0.8)	0.8 (0.7, 0.9)
**CRP (mg/L)**	1.2 (0.6, 2.8)	1.2 (0.6, 2.8)	1.5 (0.7, 3.6)
**GDF-15 (pg/mL)**	454.9 (341.8, 625.1)	487.4 (368.5, 653.6)	565.8 (425.3, 765.2)

Baseline characteristics are given as absolute and relative frequencies for categorical variables, mean ± standard deviation or quartiles (median [25^th^ percentile, 75^th^ percentile]) for continuous variables. The baseline survey was carried in 1982–84 (round 1). The cohort was re-examined in1987-1988 (round 2) and 1993–1994 (round 3). BMI = body mass index, HDL = high-density lipoprotein, LDL = low-density lipoprotein, BP = blood pressure, eGFR = estimated glomerular infiltration rate, CRP = C-reactive protein, GDF-15 = Growth differentiation factor-15.

*Number of individuals after multiple imputation

The mean age on examination was 45.5 (standard deviation: 11.0 years (51.3% male). The median follow-up was 27.51 years for death from CHD und 27.42 years for HF. Median GDF-15 serum levels at baseline were 454.9 pg/ml (25th percentile: 341.8 pg/ml; 75th percentile: 625.1 pg/ml).

### Association of single baseline measurements of GDF-15, Cystatin C and CRP with death from CHD or incidence of HF

**Fig 1:**
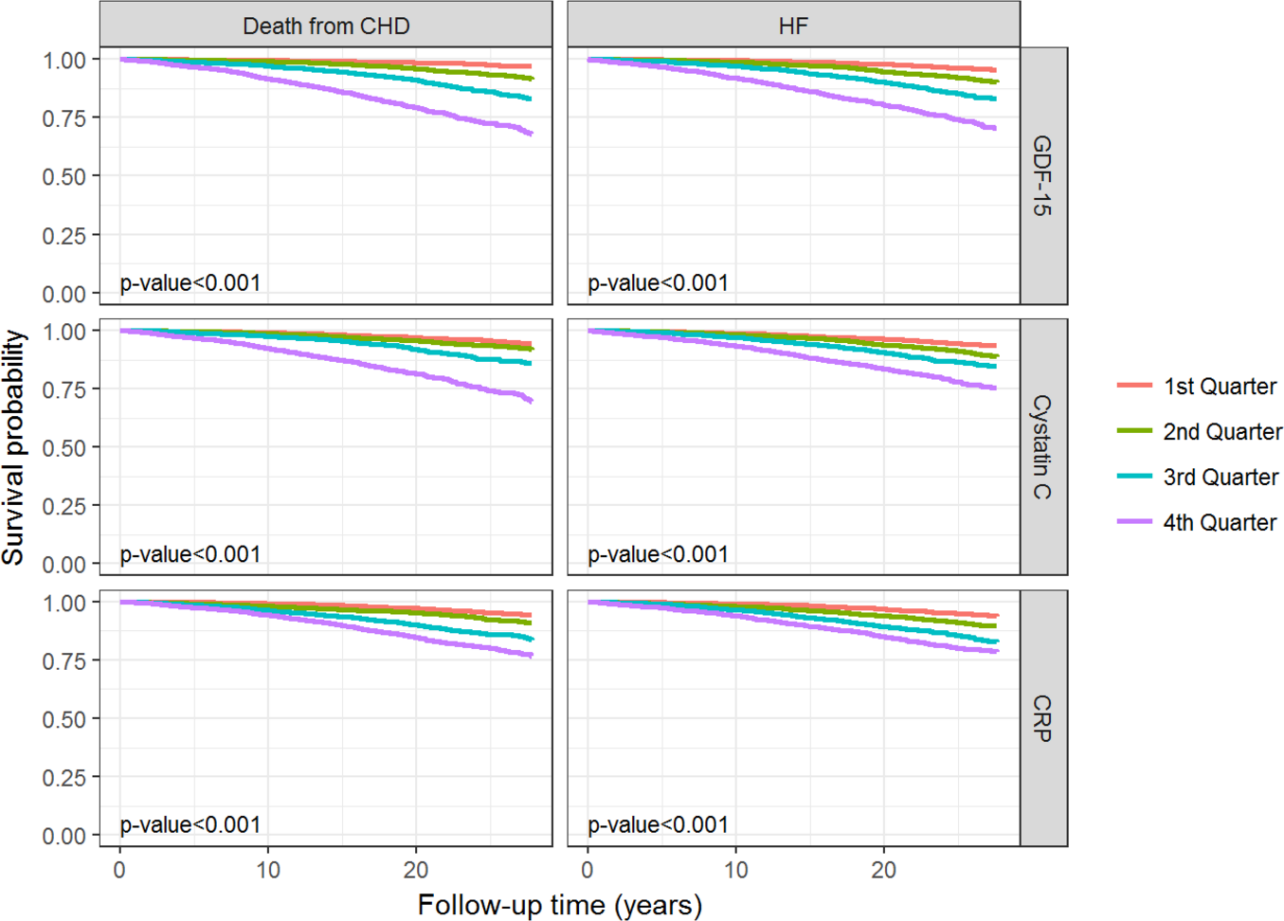
Survival curves for the endpoints death from CHD and incidence of HF. Survival curves for the endpoints death from CHD and incidence of HF according to the biomarker quarters. The p-value shown is for the logrank test.

Kaplan-Meier curves (Fig 1) reflect the difference in event-free survival for the endpoints death from CHD or incidence of HF according to the quartiles of GDF-15, CRP and Cystatin C. Kaplan-Meier curves showed increasing event rates for both endpoints with increasing GDF-15 levels for a median follow-up period of 27.53 years. These Kaplan-Meier curves indicated a higher risk of death from CHD or incidence of HF with higher GDF-15 levels.

**Fig 2:**
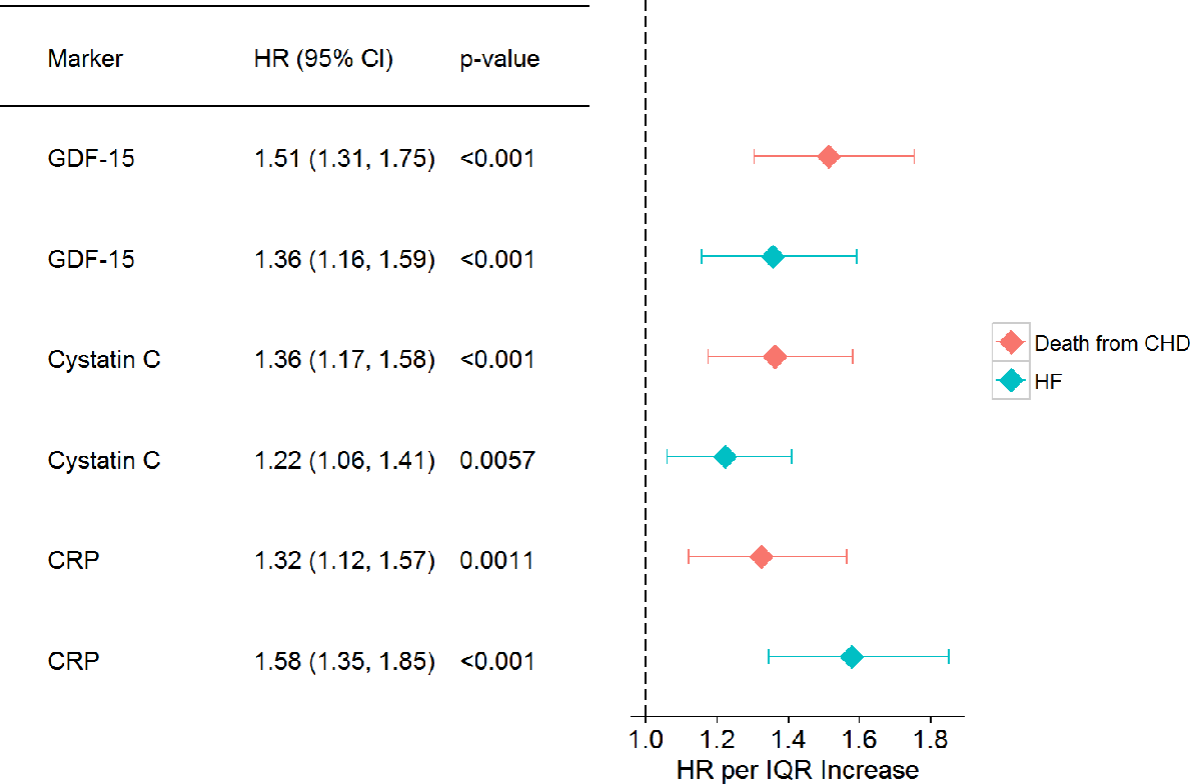
Biomarker hazard ratios for the endpoints death from CHD and incidence of HF. Cox models were adjusted for age (as the time scale), sex, overweight (BMI > 25 kg/m^2^), systolic blood pressure, diabetes, daily smoker, renal insufficiency (eGFR > 60 ml/min or 1,73m^3^). The biomarkers were used after being log-transformed. The follow-up time begins at round 1. Only round 1 measurements are used. IQR: interquartile range.

Cox regression models revealed significantly increased hazard ratios (HR) for GDF-15 for incident HF (1.36 per IQR increase, 95% confidence interval (CI): 1.16; 1.59) and death from CHD (1.51 per IQR increase, 95% CI: 1.31, 1.75) (both with p-value <0.001) (Fig 2).

### Comparison of prediction models based on the Cox regression with C-statistics

To assess the discriminative strength of the prediction models, C-indices were calculated (Table 2). Comparing the C-indices of the models with the biomarker GDF-15 to the base models for HF 0.819 (95% CI: 0.788; 0.850) and for death from CHD 0.838 (95% CI: 0.807; 0.869), the p-value for death from CHD was significant (p = 0.0097). For comparison, we fitted models with CRP and Cystatin C. The predictive value was significant only for GDF-15 according to death from CHD, while CRP was predictive for HF. C-index improvements for each biomarker alone was modest. The C-indices for the models including all three biomarkers (GDF-15, CRP and Cystatin C) to predict HF (0.824; 95% CI: 0.793, 0.855, p = 0.018) and death from CHD (0.839; 95% 0.808, 0.870, p = 0.011) were significant.

**Table 2:** C-indices for 25-year prediction of death from CHD and HF.

Death from CHD	C-index (95% CI)	C-index differences (95% CI)	p-value
Base model	0.832 (0.802, 0.863)	-	-
GDF-15 model	0.838 (0.807, 0.869)	0.00582 (0.00141, 0.01022)	0.0097
CRP model	0.834 (0.803, 0.865)	0.00166 (-0.00161, 0.00492)	0.32
CYSTATIN C model	0.836 (0.805, 0.867)	0.00371 (-0.00058, 0.00800)	0.090
GDF-15 + CRP + CYSTATIN C model	0.839 (0.808, 0.870)	0.00691 (0.00160, 0.01222)	0.011
**HF**			
Base model	0.817 (0.786, 0.847)	-	-
GDF-15 model	0.819 (0.788, 0.850)	0.00256 (-0.00073, 0.00584)	0.13
CRP model	0.824 (0.794, 0.855)	0.00780 (0.00254, 0.01306)	0.0037
CYSTATIN C model	0.816 (0.786, 0.847)	0.00018 (-0.00382, 0.00345)	0.92
GDF-15 + CRP + CYSTATIN C model	0.824 (0.793, 0.855)	0.00719 (0.00126, 0.01313)	0.018

The 25-year predicted probabilities are based on Cox models. The base model is based on the following predictors: age, sex, overweight (BMI > 25 kg/m^2^), systolic blood pressure, diabetes, daily smoker, renal insufficiency (eGFR > 60 ml/min or 1.73m^3^). The biomarkers are added to the base model. The follow-up time begins at round 1. Only round 1 measurements are used.

### Serial measurement vs. single measurement of GDF-15: Evaluation of repeated measurements using deltas and joint modeling

To evaluate if serial measurement of GDF-15 may be more useful than the most recent measurement of GDF-15, the change from round 2 (round 1 respectively) to round 3 was calculated. For GDF-15 no association could be shown with time to event (death from CHD, HF) for the changes on top of the information provided by the most recent measurement (Table 3). The CRP changes from round 1 to round 3 were associated with HF and the Cystatin C changes from round 2 to round 3 with death from CHD (both on top of the last marker measurement). The results for CRP and Cystatin C can be found in Table 3. The changes did not improve the C-index of the model with the most recent measurement for any of the markers (results not shown).

**Table 3:** Hazard ratios for Cox models with follow-up starting at round 3.

Death from CHD		
GDF-15	HR per IQR increase (95% CI)	p-value
round 3	1.99 (1.57, 2.53)	<0.001
delta round 3—round 1	1.01 (0.85, 1.2)	0.93
delta round 3—round 2	1.03 (0.83, 1.29)	0.76
**CRP**		
round 3	1.66 (1.31, 2.12)	<0.001
delta round 3—round 1	0.95 (0.77, 1.16)	0.61
delta round 3—round 2	0.93 (0.73, 1.19)	0.58
**Cystatin C**		
round 3	1.5 (1.23, 1.83)	<0.001
delta round 3—round 1	0.91 (0.79, 1.05)	0.18
delta round 3—round 2	1.25 (1.1, 1.43)	<0.001
**HF**		
**GDF-15**		
round 3	1.74 (1.38, 2.29	<0.001
delta round 3—round 1	1.16 (0.99, 1.36)	0.066
delta round 3—round 2	1.21 (0.97, 1.5)	0.085
**CRP**		
round 3	1.43 (1.14, 1.8)	0.0023
delta round 3—round 1	0.83 (0.69, 0.99)	0.043
delta round 3—round 2	0.85 (0.7, 1.05)	0.13
**Cystatin C**		
round 3	1.1 (0.89, 1.36)	0.37
delta round 3—round 1	1.06 (0.93, 1.2)	0.40
delta round 3—round 2	1.09 (0.91, 1.29)	0.35

Hazard ratios are presented for the round 3 measurement of GDF-15, CRP and Cystatin C and for the differences (delta) between rounds 3 and 1 and round 3 and 2. GDF-15, CRP and Cystatin C included used after log-transformation. The models adjusted for age, sex, overweight (BMI > 25 kg/m^2^), systolic blood pressure, diabetes, daily smoker and renal insufficiency (eGFR > 60 ml/min or 1.73m^3^). The models including a delta are additionally adjusted for the corresponding marker round 3 measurement. By “delta round 3 –round i”, i = 1 or 2, it is meant the (absolute) difference of the marker values at rounds 3 and i, that is, marker value at round 3 minus marker value at round i.

Joint modeling was used to evaluate the association of the longitudinal history of GDF-15 on the endpoints death to CHD or incident of HF (Table 4 and Table 5). This approach attempts to capture the underlying longitudinal development of the marker free of measurement error and estimates the association of the error-free marker with time-to-event [23]. The longitudinal measurements of GDF-15 were positively associated with death of CHD (HR per IQR 3.02 95% CI: (2.26, 4.04), p < 0.001) and HF (HR per IQR 2.12 95% CI: (1.54, 2.92), p < 0.001). Cystatin C and CRP showed also a positive association but smaller HRs for CHD but without a significant association for Cystatin and HF (Tables 4 and 5).

**Table 4:** Longitudinal biomarker measurements: Hazard ratios for death from CHD estimated by joint models.

	HR per IQR (95% CI)	p-value
**GDF-15**	3.02 (2.26, 4.04)	<0.001
**CRP**	2.69 (1.88, 3.84)	<0.001
**Cystatin C**	1.59 (1.3, 1.93)	<0.001

The interquartile range (IQR) of the round 1 biomarker measurement is used. Biomarkers are used after log-transformation. The hazard ratio is derived from the proportional hazards model part of the joint model and it measures the association of the time-course of the corresponding biomarker with time to death from CHD. The proportional hazards model part of the joint model was adjusted for age, sex, overweight (BMI > 25 kg/m^2^), systolic blood pressure, diabetes, daily smoker and renal insufficiency (eGFR > 60 ml/min or 1.73m^3^).

**Table 5:** Longitudinal biomarker measurements: Hazard ratios for incident HF estimated by joint models.

	HR per IQR (95% CI)	p-value
**GDF-15**	2.12 (1.54, 2.92)	<0.001
**CRP**	2.27 (1.64, 3.15)	<0.001
**Cystatin C**	1.14 (0.91,1.45)	0.25

The interquartile range (IQR) of the round 1 biomarker measurement is used. Biomarkers are used after log-transformation. The hazard ratio is derived from the proportional hazards model part of the joint model and it measures the association of the time-course of the corresponding biomarker with time to HF. The proportional hazards model part of the joint model was adjusted for age, sex, overweight (BMI > 25 kg/m^2^), systolic blood pressure, diabetes, daily smoker and renal insufficiency (eGFR > 60 ml/min or 1.73m^3^).

## Discussion

The present study revealed that a single measurement of GDF-15, independent of classical cardiovascular risk factors, seems to enhance risk prediction of death from CHD and HF compared to the biomarkers CRP and Cystatin C in the general population of the DAN-MONICA cohort. In the joint modeling of the longitudinal measurements of the biomarkers and the study endpoints, there was a stronger positive association between GDF-15 and the hazards of CHD death and HF than for single biomarker measurements. However, using GDF-15 differences of the most recent measurement with previous measurements no association could be shown and no significant improvement in discrimination was observed over that provided by the last measurement. In summary, in our study GDF-15 measurements over the time only slightly improved risk prediction for the development of HF and death due to CHD.

In the general population, the Women´s Health Study in 2002, a prospective, nested, case-control study, was the first study which revealed that increased plasma levels of GDF-15 were associated with a higher risk of CVD events in healthy women [14]. In detail, the risk of future CVD events remained after adjustment for established cardiovascular risk factors and was additive to CRP as an inflammatory biomarker [14]. Further clinical studies observed that GDF-15 was independently associated with increased cardiovascular mortality [15, 16]. In the Rancho Bernardo Study, a study with almost only middle to upper class Caucasians, elevated plasma levels of GDF-15 were powerful predictors of all-cause mortality and cardiovascular and non-cardiovascular mortality and provided prognostic information beyond proBNP and CRP [15]. Accordingly, but in a younger multiethnic cohort with 3,291 participants, Rohatgi and colleagues showed in multivariable models adjusted for conventional risk factors that increased GDF-15 concentrations (≥ 1,800 ng/L) were associated with cardiovascular mortality (HR 2.5; 95% CI 1.1–5.8, p = 0.03). Similar to the Rancho Bernardo Study these findings remained after adjustment for CRP, troponin T and NT-proBNP [15, 16]. In line with these findings, our results confirmed that single measurement of GDF-15 seems to be an independent predictor for death due to CHD among subjects from the general population. Furthermore, we showed that the measurement of GDF-15 did improve risk assessment for developing HF. Consistent to our findings, the Framingham Heart study first revealed strong association of GDF-15 levels and an increased risk for the development of HF [18]. Interestingly, in this community-dwelling cohort with 3,428 participants GDF-15 as well as the other biomarkers soluble ST2 (sST2) and high-sensitivity troponin I (hsTnI) did not predict coronary artery disease events, but total mortality [18]. Notable, in some of the previous described community-dwelling cohorts participants were elderly individuals with a higher-risk of developing CVD events and HF [15, 17, 26]. In comparison, the Dan-MONICA cohort represents a middle-aged cohort which is more similar to the Dallas Heart Study [16]. In contrast to our study population, which merely represents a northern European cohort at that time, the strength of the Dallas Heart Study is the representation of different ethnic backgrounds as mentioned above [16, 20].

Our results elucidated that repeated measurements of GDF-15, using the differences between the biomarkers according to the measurements rounds, did not significantly improve prediction of death from CHD or development of HF compared to single measurement. To further evaluate the association of multiple measurements of GDF-15 over a 27-year period with the end-points death to CHD or incidence of HF, we performed joint modeling for the biomarkers GDF-15, Cystatin C and CRP in the DAN-MONICA cohort. In our analyses, we chose to use joint models instead of the more widely used Cox regression with time dependent covariates, because the latter may underestimate the magnitude of the association of the evolution in time of the biomarker and time-to-event [23, 24, 27]. The longitudinal evolution of the biomarkers via joint modeling showed a stronger positive association of GDF-15 with death from CHD and HF than the single biomarker measurements. Related to our findings, Eggers et al. found that baseline concentrations of GDF-15 could predict future CVD events and especially all-cause mortality in an elderly community-dwelling population [26]. In detail, changes over the time of GDF-15 predicted individual´s all-cause mortality, whereas GDF-15 and cardiovascular events displayed no significant association [26].

In population-based studies, CRP as a marker for inflammation and Cystatin C as marker for renal dysfunction have also been described to be associated with risk of future CVD [3, 28–33]. Shlipak and colleagues first reported that Cystatin C was an independent predictor of death from cardiovascular causes and superior to creatinine in an elderly population of the community [29]. Numerous follow-up studies confirmed the role of Cystatin C as a predictor of cardiovascular death in the general population [28, 30, 34]. Our data agree with these findings, that Cystatin C is a strong predictor of cardiovascular death. Nonetheless, Cystatin C, in our cohort has no prognostic value for future HF. Previous studies have noted the association of CRP with cardiovascular mortality and development of HF in community-dwelling individuals [31–33, 35]. Our results also confirm other studies supporting that CRP is an independent predictor for cardiovascular mortality in community-dwelling individuals. Moreover, Zethelius et al. observed in a community-based elderly population that the addition of several biomarkers (Troponin I, NT-proBNP, Cystatin C and high-sensitivity CRP) to traditional cardiovascular risk factors improved risk stratification for cardiovascular death [36]. In this context, our data also indicate that an including CRP measurement, and especially GDF-15 measurement, improves risk estimation of HF and death from CHD over that of cardiovascular risk factors.

### Limitations and strengths

This study has several limitations and strengths. One major strength of the The DAN-MONICA project is a large-scale and population-based cohort with more than 3,500 persons. In addition, the long-term follow-up period of more than 27 years allows a reliable longitudinal analysis. Nevertheless, this northern European cohort does not reveal possible ethnic differences in the results, which might withhold an immediate transfer into other populations. Unfortunately, data on NT-proBNP are not available in the DAN-MONICA cohort, therefore a comparison to NT-proBNP as a standard biomarker for HF risk stratification is not available. In addition, we cannot exclude the possibility that storage at -20°C the early years and, at -80°C since 2010 might have affected the measured biomarker levels as a result of potential sample degradation or evaporation of water.

## Conclusion

GDF-15 seems to be a promising biomarker for prediction of HF and death due to CHD in the general population, which may enhance prognostic information to established clinical biomarkers. Repeated measurements of GDF-15 displayed only a slight improvement in risk prediction of these endpoints compared to single measurement. Prospective and randomized clinical studies are needed to evaluate the utilization of GDF-15 in combination or comparison to other biomarkers in the clinical setting.

## Supporting information

S1 File Statistical analysis. (PDF)

S1 Table Baseline characteristics of the DAN-MONICA cohort according to round 1–3 (Data for the nonimputed datasets). Baseline characteristics are given as absolute and relative frequencies for categorical variables, mean ± standard deviation (mean±standard deviation) or quartiles (median (25^th^ percentile, 75^th^ percentile) for continuous variables. The baseline survey was carried in 1982–84 (round 1). The cohort was re-examined in1987-1988 (round 2) and 1993–1994 (round 3). BMI = body mass index, HDL = high-density lipoprotein, LDL = low-density lipoprotein, BP = blood pressure, eGFR = estimated glomerular infiltration rate, CRP = C-reactive protein, GDF-15 = Growth differentiation factor-15.
(PDF)

S2 Table C-indices for 5-year prediction of death from CHD and HF. The 5-year predicted probabilities are based on Cox models. The base model is based on the following predictors: age, sex, overweight (BMI > 25 kg/m^2^), systolic blood pressure, diabetes, daily smoker, renal insufficiency (eGFR > 60 ml/min or 1.73m^3^). The biomarkers are added to the base model. The follow-up time begins at round 1 and only the first 5 years of follow-up were used. Only round 1 measurements are used. 31 deaths from CHD and 25 HF cases were observed during the 5 years´ follow-up used.
(PDF)

S3 Table C-indices for 10-year prediction of death from CHD and HF. The 10-year predicted probabilities are based on Cox models. The base model is based on the following predictors: age, sex, overweight (BMI > 25 kg/m^2^), systolic blood pressure, diabetes, daily smoker, renal insufficiency (eGFR > 60 ml/min or 1.73m^3^). The biomarkers are added to the base model. The follow-up time begins at round 1 and only the first 10 years of follow-up were used. Only round 1 measurements are used. 77 deaths from CHD and 62 HF cases were observed during the 10 years´ follow-up used.
(PDF)
