# Supplementary material for: Predictive value of long-term changes of growth differentiation factor-15 over a 27-year-period for heart failure and death due to coronary heart disease
Source: PLoS One. 2018 May 17;13(5):e0197497. doi: 10.1371/journal.pone.0197497 (PMC5957420; doi:10.1371/journal.pone.0197497)
Supplement: S1 File — (PDF) [file pone.0197497.s001.pdf]

Statistical analysis

Baseline characteristics are given as absolute and relative frequencies for categorical variables, mean and standard deviation or quartiles for continuous variables.

Multiple imputation was used to deal with missing data. Twenty imputed datasets were produced using chained equations (15). Predictive mean matching was used for all variables and time-to-event information was incorporated into the imputation model via the Nelson-Aalen estimator of the cumulative hazard function. For the imputation the data were used in the wide format, meaning that for each variable that was measured at each examination round, the data contained 3 columns (one for each examination). Separate imputations were produced for men and women.

First, the association of the round 1 measurements of the biomarkers of interest (GDF-15, CRP and Cystatin C) with death due to CHD and incident HF was examined. The biomarkers were categorized using their quartiles and survival curves were produced by the Kaplan-Meier method. The logrank test was performed to compare the survival curves defined by the biomarker quarters. Afterwards, Cox regressions adjusted for sex, overweight (BMI > 25 kg/m²), systolic blood pressure, diabetes, daily smoking, renal insufficiency (eGFR < 60 mL/min for 1.73m²) were computed. Age was used as the time scale in the Cox models. To quantify the discrimination of the 25-year event probabilities, derived from these Cox models, the C-index was computed. To obtain these probabilities the Breslow estimator of the baseline survival function was used. 10-fold cross-validation was used to avoid over-optimism resulting from assessment of the model performance on the same data in which the model was derived (16) .

To examine the association of outcome to the longitudinal measurements of the biomarkers, a joint model for the longitudinal marker and time to event was computed (17). The joint model consists of two parts, a linear mixed effect model for the biomarker and a proportional hazards model for the survival part. For each marker the linear mixed effect model included a random intercept and a

random slope from time since baseline for each individual. Sex, age at round 1 (cross-sectional effect of age), time since baseline (longitudinal effect of age), overweight, systolic blood pressure, LDL cholesterol, diabetes, daily smoker and renal insufficiency were used as fixed effects in the mixed effect model. The survival part of the joint model used age as the time scale and was adjusted for the same covariates as was the Cox model described in the previous paragraph. For the latter model the values of the covariates were updated at each round. The following two equations describe both submodels for the ith individual and how they are related, observe that *t* denotes the ith individual age:

$$\begin{aligned}\log(\text{marker})(t) = \quad & \beta_0 + b_{i0} + (\beta_1 + b_{i1}) \cdot \text{time since baseline}(t) + \beta_3 \cdot \text{Age at baseline} \\ & + \beta_4 \cdot \text{sex} + \beta_5 \cdot \text{overweight}(t) + \beta_6 \cdot \text{systolic blood pressure}(t) \\ & + \beta_7 \cdot \text{LDL cholesterol}(t) + \beta_8 \cdot \text{diabetes}(t) + \beta_9 \cdot \text{daily smoker}(t) \\ & + \beta_{10} \cdot \text{renal insufficiency}(t) + \epsilon_i(t) = m_i(t) + \epsilon_i(t)\end{aligned}$$

$$\begin{aligned}h_i(t) = \quad & h_0(t) \cdot \exp\{\alpha \cdot m_i(t) + \gamma_1 \cdot \text{sex} + \gamma_2 \cdot \text{overweight}(t) + \gamma_3 \cdot \text{systolic blood pressure}(t) \\ & + \gamma_4 \cdot \text{LDL cholesterol}(t) + \gamma_5 \cdot \text{diabetes}(t) + \gamma_6 \cdot \text{daily smoker}(t) \\ & + \gamma_7 \cdot \text{renal insufficiency}(t)\}\end{aligned}$$

where h_i is the hazard function at age *t*; $\epsilon_i(t)$ are normally distributed with mean 0 and standard deviation σ; the β's are fixed effects; b_{i0}, b_{i1} are random effects having a joint centered normal distribution with covariance matrix D. B-splines are used in the estimation of the logarithm of the baseline hazard function $h_0(t)$. The random effects b_{i0} and b_{i1} are shared by both submodels and account for the correlation of the repeated marker measurements and for the association of the marker with time-to-event. The marker and time-to-event are assumed to be independent given the shared random effects. All parameters are estimated by maximization of the joint distribution likelihood of time-to-event and the longitudinal marker.

To assess possible improvement for prediction by information on past changes in biomarker values, the available marker history up to the third examination (round 3) was modelled in different ways. The difference between the values at round 3 and round 2 (round 1 respectively) was considered and the round 3 measurement was also used. These variables entered a Cox model with follow-up time starting at round 3. All models used the same adjusted covariates as the Cox regressions described earlier and age was used as the time scale. The models including the marker differences as predictors

where further adjusted for the round 3 measurement of the corresponding marker. The C-index was used quantify the gain of the marker history over the last available marker measurement (at round 3).

For all regression analyses the biomarkers were used after being log-transformed. Analyses were performed with R version 3.3.0 (18)
