# Supplementary material for: Predictive value of long-term changes of growth differentiation factor-15 over a 27-year-period for heart failure and death due to coronary heart disease
Source: PLoS One. 2018 May 17;13(5):e0197497. doi: 10.1371/journal.pone.0197497 (PMC5957420; doi:10.1371/journal.pone.0197497)
Supplement: S1 Table — Baseline characteristics are given as absolute and relative frequencies for categorical variables, mean ± standard deviation (mean±standard deviation) or quartiles (median (25th percentile, 75th percentile) for continuous variables. The baseline survey was carried in 1982–84 (round 1). The cohort was re-examined in1987-1988 (round 2) and 1993–1994 (round 3). BMI = body mass index, HDL = high-density lipoprotein, LDL = low-density lipoprotein, BP = blood pressure, eGFR = estimated glomerular infiltration rate, CRP = C-reactive protein, GDF-15 = Growth differentiation factor-15. (PDF) [file pone.0197497.s002.pdf]

S1 Table. Baseline characteristics of the DAN-MONICA cohort according to round 1-3 (Data for the nonimputed datasets).

	round 1 (n=3785)	round 2 (n=2987)	round 3 (n=2656)
**Examination age (years)**	45.5±11	50.4±11	55.4±10.8
**Male (%)**	1940 (51.3)	1524 (51)	1289 (50.5)
**BMI (kg/m²)**	24.6±3.9	25.1±4	25.8±4.2
**BMI>25 kg/m² No. (%)**	1524 (40.3)	1360 (45.5)	1343 (52.6)
**HDL cholesterol (mmol/L)**	1.5±0.4	1.5±0.4	1.4±0.4
**LDL cholesterol (mmol/L)**	3.7±1.1	4±1.1	4.1±1
**Total cholesterol (mmol/L)**	5.8±1.2	6.1±1.2	6.2±1.1
**Systolic BP (mmHg)**	123.3±16.8	126.4±18.8	129.2±19
**Diastolic BP (mmHg)**	77.2±10.9	81.7±10.5	82±10.5
**Daily smoker No. (%)**	1768 (46.7)	1417 (48.9)	937 (36.7)
**Diabetes No. (%)**	86 (2.3)	92 (3.1)	89 (3.5)
**eGFR (mL/min for 1.73m²)**	110.1 (97.0, 121.5)	104.7 (93.1, 114.3)	98.0 (86.1, 108.0)
**eGFR<60 mL/min for 1.73m² No. (%)**	76 (2)	41 (1.5)	65 (2.6)
**Creatinine (mg/dL)**	0.8 (0.7, 0.9)	0.8 (0.7, 0.9)	0.85 (0.8, 1.0)
**Cystatin C (mg/L)**	0.7 (0.6, 0.8)	0.73 (0.6, 0.8)	0.77 (0.7, 0.9)
**CRP (mg/L)**	1.2 (0.6, 2.81)	1.2 (0.5, 2.7)	1.45 (0.7, 3.3)
**GDF-15 (pg/mL)**	456 (342.0, 625.7)	480 (365.2, 642.1)	551.7 (419.0, 739.9)

Baseline characteristics are given as absolute and relative frequencies for categorical variables, mean ± standard deviation (mean±standard deviation) or quartiles (median (25th percentile, 75th percentile) for continuous variables. The baseline survey was carried in 1982-84 (round 1). The cohort was re-examined in 1987-1988 (round 2) and 1993-1994 (round 3). BMI= body mass index, HDL=high-density lipoprotein, LDL=low-density lipoprotein, BP=blood pressure, eGFR= estimated glomerular infiltration rate, CRP= C-reactive protein, GDF-15=Growth differentiation factor-15.
