# Supplementary material for: Predictive value of long-term changes of growth differentiation factor-15 over a 27-year-period for heart failure and death due to coronary heart disease
Source: PLoS One. 2018 May 17;13(5):e0197497. doi: 10.1371/journal.pone.0197497 (PMC5957420; doi:10.1371/journal.pone.0197497)
Supplement: S2 Table — The 5-year predicted probabilities are based on Cox models. The base model is based on the following predictors: age, sex, overweight (BMI > 25 kg/m2), systolic blood pressure, diabetes, daily smoker, renal insufficiency (eGFR > 60 ml/min or 1.73m3). The biomarkers are added to the base model. The follow-up time begins at round 1 and only the first 5 years of follow-up were used. Only round 1 measurements are used. 31 deaths from CHD and 25 HF cases were observed during the 5 years´ follow-up used. (PDF) [file pone.0197497.s003.pdf]

S2 Table. C-Indices for 5-year prediction of death from CHD and HF

Death from CHD	C-index (95% CI)	C-index differences (95% CI)	p-value
Base model	0.750 (0.646, 0.854)	-	-
GDF-15 model	0.791 (0.687, 0.895)	0.04076 (0.00814, 0.07338)	0.014
CRP model	0.759 (0.655, 0.864)	0.00911 (-0.01310, 0.03131)	0.42
CYSTATIN C model	0.762 (0.657, 0.867)	0.01152 (-0.01532, 0.03835)	0.40
**HF**			
Base model	0.783 (0.669, 0.898)	-	-
GDF-15 model	0.800 (0.686, 0.914)	0.01691 (0.00254, 0.03129)	0.021
CRP model	0.816 (0.702, 0.930)	0.03252 (-0.00065, 0.06569)	0.055
CYSTATIN C model	0.801 (0.687, 0.915)	0.01768 (-0.01328, 0.04863)	0.26

The 5-year predicted probabilities are based on Cox models. The base model is based on the following predictors: age, sex, overweight (BMI > 25 kg/m^2), systolic blood pressure, diabetes, daily smoker, renal insufficiency (eGFR > 60 ml/min or 1,73m^3). The biomarkers are added to the base model. The follow-up time begins at round 1 and only the first 5 years of follow-up were used. Only round 1 measurements are used. 31 deaths from CHD and 25 HF cases were observed during the 5 years´ follow-up used.
