# Supplementary material for: Predictive value of long-term changes of growth differentiation factor-15 over a 27-year-period for heart failure and death due to coronary heart disease
Source: PLoS One. 2018 May 17;13(5):e0197497. doi: 10.1371/journal.pone.0197497 (PMC5957420; doi:10.1371/journal.pone.0197497)
Supplement: S3 Table — The 10-year predicted probabilities are based on Cox models. The base model is based on the following predictors: age, sex, overweight (BMI > 25 kg/m2), systolic blood pressure, diabetes, daily smoker, renal insufficiency (eGFR > 60 ml/min or 1.73m3). The biomarkers are added to the base model. The follow-up time begins at round 1 and only the first 10 years of follow-up were used. Only round 1 measurements are used. 77 deaths from CHD and 62 HF cases were observed during the 10 years´ follow-up used. (PDF) [file pone.0197497.s004.pdf]

S3 Table. C-Indices for 10-year prediction of death from CHD and HF

Death from CHD	C-index (95% CI)	C-index differences (95% CI)	p-value
Base model	0.826 (0.761, 0.891)	-	-
GDF-15 model	0.848 (0.782, 0.913)	0.02131 (0.00573, 0.03689)	0.0074
CRP model	0.827 (0.762, 0.892)	0.00083 (-0.00553, 0.00720)	0.80
CYSTATIN C model	0.832 (0.767, 0.897)	0.00536 (-0.00651, 0.01723)	0.38
**HF**			
Base model	0.831 (0.759, 0.903)	-	-
GDF-15 model	0.840 (0.768, 0.912)	0.00897 (-0.00235, 0.02029)	0.12
CRP model	0.855 (0.783, 0.928)	0.02399 (0.00682, 0.04116)	0.0062
CYSTATIN C model	0.838 (0.766, 0.910)	0.00658 (-0.00830, 0.02146)	0.39

The 10-year predicted probabilities are based on Cox models. The base model is based on the following predictors: age, sex, overweight (BMI > 25 kg/m^2), systolic blood pressure, diabetes, daily smoker, renal insufficiency (eGFR > 60 ml/min or 1,73m^3). The biomarkers are added to the base model. The follow-up time begins at round 1 and only the first 10 years of follow-up were used. Only round 1 measurements are used. 77 deaths from CHD and 62 HF cases were observed during the 10 years´ follow-up used.
